# Immunotherapy for asthma

**DOI:** 10.1038/s41423-025-01357-9

**Published:** 2025-10-27

**Authors:** Hamida Hammad, Engi Ahmed, Bart N. Lambrecht

**Affiliations:** 1 Laboratory of Immunoregulation and Mucosal Immunology, VIB-UGent Center for Inflammation Research, Ghent, Belgium; 2 Department of Internal Medicine and Pediatrics, Ghent University, Ghent, Belgium; 3 Department of Pulmonary Medicine, University of Montpellier, INSERM U1046, CNRS UMR 9214. Hôpital Arnaud de Villeneuve CHU, Montpellier, France

**Keywords:** Asthma, endotypes, allergens, immunotherapy, biologics., Immunology, Inflammation, Mechanisms of disease

## Abstract

Type 2^high^ asthma, which accounts for the majority of asthma cases, is driven by Th2 cells that produce cytokines such as IL-4, IL-5, and IL-13. These cytokines promote several features of the disease, including eosinophilia, IgE production, bronchial hyperresponsiveness (BHR), mucus hypersecretion, and susceptibility to exacerbations. In contrast, type 2^low^ asthma is characterized by the presence of neutrophils and reduced responsiveness to corticosteroids. In recent years, advances in our understanding of the distinct mechanisms at play in each asthma endotype have paved the way for the development of targeted therapies tailored to specific patient profiles. In this review, we first explore the underlying immunological mechanisms of various asthma endotypes. We also provide an overview of the different types of immunotherapies currently available to asthmatic patients and their clinical efficacy. Finally, we highlight emerging therapeutic strategies that hold promise for improving asthma management in the future.

## Main text

Asthma is a chronic inflammatory disease of the lower airways that affects approximately 260 million people worldwide [1]. This disease accounts for 0.5 million deaths per year, and its prevalence continues to increase. Asthma is characterized by wheezing, cough, shortness of breath, and bronchial hyperresponsiveness (BHR). In more severe forms of the disease, patients present mucus plugging that contributes to chronic airway obstruction and therefore also to the severity of the disease [2]. The pathogenesis of asthma is complex and not completely understood, and the disease can be further complicated by respiratory virus infections or environmental factors that are responsible for asthma exacerbation [3]. Although a wide range of treatments is available, some patients still experience poorly controlled disease, which requires frequent hospitalizations or emergency room visits.

Asthma often begins in childhood (childhood-onset asthma) and is often associated with exaggerated type 2 immunity, involving the production of the prototypical type 2 cytokines IL-4, IL-5, and IL-13. However, some patients develop asthma later in life, the latter being referred to as late-onset asthma. Childhood-onset asthma and late-onset asthma differ at many levels. In children, allergic sensitization mostly drives a response dominated by eosinophils. In contrast to childhood-onset asthma, adult-onset asthma is more severe and less strongly associated with atopy. Patient-specific differences in age of onset, degree of severity, comorbidities, associated risk factors, and response to treatment have allowed us to appreciate the complexity of asthma [4]. Airflow obstruction that is reversible, either spontaneously or following treatment with bronchodilators or corticosteroids, is a common feature of asthma. It is now clear that only a proportion of asthma patients have reversible airway obstruction and that some of them have persistent airflow obstruction caused by the presence of tenacious mucus plugs. It has been reported that such mucus deposits are common in fatal asthma cases and can be present in up to 70% of patients with severe forms of asthma [5, 6].

**Fig. 1:**
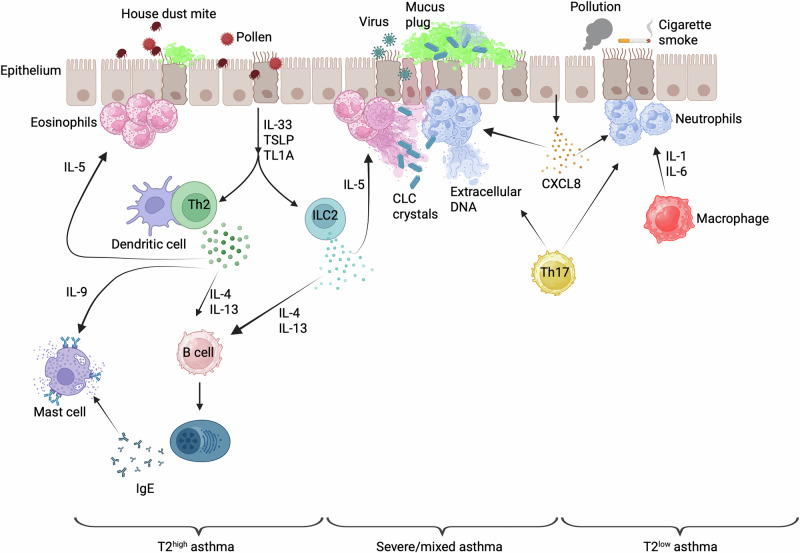
Immune mechanisms underlying the different asthma endotypes

For several years, clinicians have recognized asthma as a heterogeneous disease with different endotypes. Endotypes are defined by underlying pathophysiological mechanisms that might lead to differences in the way patients respond to common therapies, such as inhaled corticosteroids or specific biologicals [7–9]. Two major endotypes have been described thus far: the type 2-high or type 2-ultrahigh endotype, which is essentially eosinophilic, and the type 2-low endotype, which is noneosinophilic, sometimes neutrophilic, and metabolic [10]. In most cases, especially in children, the T2^high^ endotype is dominated by type 2 immunity-associated cytokines and a high number of eosinophils and responds well to inhaled corticosteroids and bronchodilators [11] (Fig. 1). Some severe adult-onset T2^high^ asthma patients can present with comorbidities such as rhinosinusitis and/or obesity, and their disease is more steroid resistant. The type 2-low endotype is more complex, as it generally includes all asthmatic patients with no type 2-high inflammation, in which no biomarkers have been identified. The pathophysiology of type 2^low^ asthma is currently unclear, but it seems to be associated with a more severe form of the disease and with an unresponsiveness to glucocorticoids. Type 2^low^ asthma can be dominated by an IL-17 response, and the European Severe Asthma Network has revealed an IL-17 signature in epithelial cells of approximately 25% of patients with asthma. These patients are characterized by sputum neutrophilia and frequent exacerbations [12] (Fig. 1). Additionally, high levels of IL-1, IL-8, IL-17, TNFα, or GM-CSF have been detected in the bronchoalveolar lavages of patients with type 2^low^ asthma [13]. Furthermore, type 2^low^ patients can also experience mixed neutrophilic and eosinophilic inflammation associated with Th1-like inflammation dominated by high levels of IFN-γ [14, 15]. In addition, some patients can develop paucigranulocytic asthma, a form of asthma without elevated eosinophils or neutrophils. Several studies have reported that paucigranulocytic asthma is the most common inflammatory phenotype (reviewed in [16]), although these data might be biased by the different cutoff values used in the studies. The mechanisms driving paucigranulocytic asthma remain largely unknown, but transcriptomics data point to an upregulation of genes involved in metabolism and in mitochondrial function [17] along with elevated counts of sputum macrophages and mast cells [18, 19].

Despite all the knowledge gained over the years from transcriptomics data, asthma remains a very complex and heterogeneous disease. Even if patients with asthma can be classified into 2 endotypes, the disease in patients with the same endotype involves different mechanisms. It is therefore unthinkable that a single treatment could benefit all patients. A better understanding of the mechanisms at play in each patient has allowed the development of a battery of targeted approaches to control the course of the disease. In this review, we discuss the main mechanisms involved in the different asthma endotypes. We also provide an overview of the different therapeutic options currently available to patients and their therapeutic efficacy, and discuss some promising future strategies for asthma management.

## Immunology for asthma

### T2^high^ asthma

If we want to offer the best tailored treatment to patients with asthma, it is important to understand the mechanisms underlying both T2^high^ and ^T2low^ asthma.

In T2^high^ asthma (Fig. 1, left), the epithelium plays a central role in asthma pathogenesis. The epithelium is considered more than a physical barrier between the lung and the outside world. It can actively orchestrate immune responses by producing chemokines and cytokines, called alarmins, which can recruit and activate several immune cells, such as eosinophils, dendritic cells (DCs), lymphocytes, and innate lymphoid cells (ILCs) [20]. In response to allergens, stressed and/or damaged epithelial cells can release alarmins such as TSLP, IL-1α, IL-33, and GM-CSF, which can directly activate IRF4^+^ type 2 conventional dendritic cells (cDC2s) to migrate to the lung-draining lymph node. The migratory cDC2s that reach the draining nodes stimulate the differentiation of naïve T cells into effector Th2 cells [21–23]. Recently, Th2 differentiation in the lymph nodes was shown to occur in large clusters of T cells interacting with migratory DCs [23].

Th2 cells are considered pathogenic and drivers of eosinophilic airway inflammation. Once they enter the lung, Th2 cells are activated locally by different signals derived from epithelial cells (mainly alarmins), which determine the cytokine profile that is released by T cells [24]. Th2 cells are pathogenic in asthma because they produce high amounts of the prototypical type 2 cytokines IL-4, IL-5, IL-9, and IL-13. IL-5 induces the maturation of bone marrow eosinophil progenitors and contributes to the survival of eosinophils in the lung [25]. IL-4 and IL-13 control cellular recruitment, allergen-specific IgE production, mucus metaplasia, and airway hyperresponsiveness [26]. IL-9 promotes the survival and proliferation of histamine-producing mast cells and contributes to several features of asthma, including airway hyperresponsiveness [27–29]. Although Th2 cells are at the heart of asthma pathogenesis, group 2 innate lymphoid cells (ILC2s) have gained interest in recent years because of their ability to produce type 2 cytokines and because they can be activated by the epithelial-derived alarmins IL-33, IL-25, and TSLP (Fig. 1, left). Recent work has shown that another epithelial-derived alarmin, called TL1A, can act with IL-33 to increase IL-9 production by ILC2s, which then becomes pathogenic, suggesting that IL-9 might represent an interesting target in asthma [27, 30].

### T2^low^ asthma

Although the pathophysiology of T2^low^ asthma is not yet fully understood, the airway inflammation that develops in these patients seems to involve the activation of the inflammasome as well as Th1 and/or Th17 cells (Fig. 1, right). Indeed, patients with T2^low^ asthma generally have high amounts of IL-1β and neutrophils in their sputum. These neutrophils are clearly activated, as suggested by the presence of neutrophil-derived extracellular traps [31]. An underlying mechanism in T2^low^ asthma might be the presence of a Th1/Th17 signature in these patients [13, 14, 31]. Both interferon-γ (IFNγ)- and IL-17-associated gene signatures are associated with increased inflammation and airway resistance and with a reduced response to inhaled corticosteroids [32, 33]. Although the T2^low^ endotype has been shown to be linked to Th1/Th17 responses, an intriguing recent finding was that the alarmin TSLP, initially linked to T2^high^ asthma, was also present in the airways of T2^low^ asthma patients [34]. TSLP has been shown to contribute to Th17 cell polarization and, as such, could also drive neutrophilic asthma [35]. To make things even more complex, neutrophils have been shown to influence type 2-high asthma. A study reported that following rhinovirus infection, extracellular traps derived from neutrophils were able to exacerbate T2^high^ asthma [36]. In addition, patients with a more severe form of asthma usually have mixed neutrophilic and eosinophilic inflammation (Fig. 1, center) and display type 1 cytokine signatures of IFNG, STAT1, and CXCL9/10, which are associated with steroid resistance [37]. These patients have persistent asymptomatic carriage of respiratory viruses, and it is still unclear how such carriage can affect the immunological profile and severity of the disease in such patients.

## Immunotherapy for asthma

As we gain more knowledge into the underlying mechanisms in asthma, it becomes clear that the spectrum of the disease is far more complex than initially anticipated. Different endotypes have been described, and within each endotype, there is considerable heterogeneity among patients. Therefore, the use of currently available individualized strategies that fit the needs of each patient is crucial.

### Allergen-specific immunotherapy for asthma

Allergen-specific immunotherapy (AIT) consists of administering repetitive high doses of allergens for several years to achieve a disease-free state. AIT is offered to people with atopy for whom allergic rhinitis or allergic asthma is caused by allergen exposure. The first evidence of the efficacy of AIT in asthma dates from the early 50s, when grass pollen was used to treat seasonal asthma [38]. The efficacy of this approach was attributed to the protein fraction of the extract used. It was only in the late 1970s that the concept of allergen specificity for AIT was demonstrated. A study by Norman et al. was the first to show that AIT against one allergen in dual-sensitized patients could relieve symptoms induced only by the allergen used in AIT [39]. Until very recently, injections of allergens were given subcutaneously every week for several years, and the long-term benefits of AIT disease-modifying effects have been reported and confirmed by several groups [40, 41].

Although AIT has been proven to be beneficial in patients with allergic rhinitis, an early meta-analysis revealed that it also improved asthma symptoms and airway hyperresponsiveness and promoted less drug usage [42]. Indeed, when used as an add-on strategy in patients with mild to moderate asthma, subcutaneous AIT (SCIT) helps reduce the use of inhaled β2 agonists [43] and decreases type 2 cytokine release (IL5 and IL-13) by PBMCs [44]. Several studies have reported the efficacy of SCIT in patients with severe asthma without persistent airflow obstruction (reviewed in [45]). Despite the positive effect of SCIT in treating atopic diseases in general, the reliance on repetitive injections subcutaneously implies the adherence of patients to a strict injection scheme, which can become a burden for many of these patients. In addition, systemic reactions are a potential risk for SCIT. To circumvent this issue, a novel route of allergen administration has been tested, and sublingual immunotherapy (SLIT) was introduced in Europe a couple of years ago. SLIT is considered safer, easier and less painful and is now in clinical use in many countries. House dust mite (HDM)-SLIT has demonstrated clinical efficacy in improving asthma symptoms, as evidenced by a reduced reliance on inhaled corticosteroids and a lower incidence of asthma exacerbations [46–49]. SLIT is now recommended for patients with asthma complicated by allergic rhinitis, for whom inhaled corticosteroids cannot improve symptoms, and only with an FEV1 > 70% [50]. Recently, the effects of SLIT and its ability to increase lung function and decrease airway inflammation and asthma symptoms have been shown to last for 5 years, revealing the long-term effects of AIT in asthma [51].

#### Mechanisms of AIT

Given the significant effects of allergen immunotherapy (AIT) in patients with T2^high^ allergic asthma, researchers have sought to elucidate the underlying mechanisms of its efficacy. Over the years, our understanding of how AIT alleviates symptoms has progressively deepened. Sensitized patients exposed to the relevant allergen normally develop a response dominated by an exaggerated type 2 immune response. The presence of IgE and its subsequent binding to the high-affinity receptor (FcεRI) on mast cells triggers the degranulation of mast cells and basophils. In addition, the presence of epithelial-derived alarmins (IL-33, TSLP, IL-1, and GM-CSF) and type 2 cytokines (IL-4, IL-5, IL-13, and IL-9) produced by Th2 effector cells or ILC2s contributes to tissue eosinophilia, smooth muscle contraction, airway hyperresponsiveness, and tissue remodeling (reviewed in (2)).

**Fig. 2:**
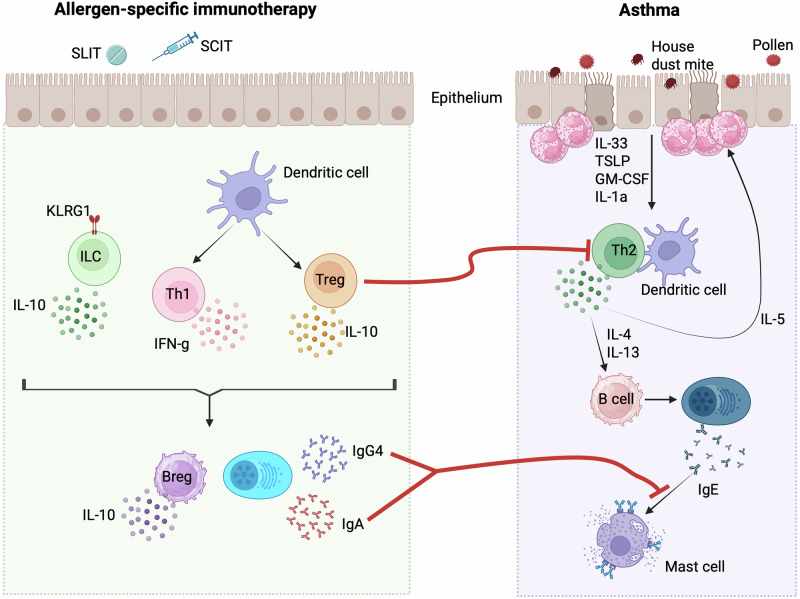
Mechanisms of action of allergen-specific immunotherapy. T2^high^ asthma is characterized by an exaggerated type 2 immune response associated with eosinophilic airway inflammation, the production of Th2-associated cytokines and allergen-specific IgE production (right). The administration of AIT (left side) has the potential to affect dendritic cell and ILC2 functions, favoring a response driven by IL-10 and Treg cells. These Tregs have the potential to block pathogenic Th2 cells, leading to symptom improvement in asthmatic patients. Additionally, AIT induces the production of IgA and IgG4, which prevent IgE-mediated mast cell degranulation

Several mechanisms have been proposed to explain the success of AIT (Fig. 2). First, AIT reportedly desensitizes mast cells and basophils through the upregulation of the inhibitory Fc receptor FcγRIIb, which in turn impairs IgE-FcεRI crosslinking and the degranulation of these cells [52]. However, to establish lasting tolerance to allergens, additional mechanisms engaging different cell types have been identified. As such, AIT has been shown to induce and maintain the function of regulatory T (Treg) and B (Breg) cells (Fig. 2). The repetitive exposure to the allergen led to the generation of IL-10-producing dendritic cells, which were able to induce and expand allergen-specific Tregs in humans [53, 54]. A recent report in a mouse model of SLIT showed that the preferential induction of Tregs, rather than Th2 cells, was mediated by cDC2s in the draining cervical lymph nodes. These cDC2s, under the influence of AIT, are rewired to induce a suppressive environment [55]. These Tregs exert their anti-inflammatory functions through various mechanisms, involving the production of inhibitory cytokines (IL-10, IL-35, or TGF-β) and the high surface expression of proteins with suppressive activity (PD-1, CTLA-4, ICO,S and LAG-3) [56]. During AIT, Tregs also contribute to the generation of blocking antibodies, with IL-10 preferentially inducing IgG4 class switching in B cells and TGFβ promoting IgA release [57, 58]. These allergen-specific immunoglobulins can compete with IgE for binding to the allergen and can interfere with FcεRI crosslinking in mast cells [59] (Fig. 2). They can also impair IgE-facilitated allergen presentation by B cells and dendritic cells, limiting or inhibiting the generation of effector pathogenic Th2 cells [59].

While AIT can influence the response of T and B cells, recent findings suggest that it can also affect cells from the innate immune system. As such, grass pollen AIT was shown to suppress the increase in ILC2 numbers normally observed during the pollen season and to regulate both their phenotype and function. Grass pollen AIT can effectively promote the generation of KLRG1^+^ IL-10^+^ ILC2s, which can dampen inflammatory responses to allergens through a mechanism that involves retinoic acid [60, 61] (Fig. 2). In addition, one year after subcutaneous AIT, the composition of monocytes and dendritic cells in the blood of patients changes [62]. AIT induces an enrichment of monocytes with anti-inflammatory properties, characterized by increased IL-10 expression and reduced expression of TNF-α and IL-1β. At the dendritic cell level, AIT increases the number of pDCs and decreases the number of CD1c^±^ DCs in the circulation [62]. Since pDCs were shown in mice to be immunoregulatory [63, 64] and cDCs were shown to support Th2 responses [65], the changes in the DC balance induced by AIT might explain why AIT induces potent regulatory responses at the expense of type 2 immunity.

The fact that AIT, which is based on repetitive exposure to an allergen, can affect the innate immune compartment has recently raised the possibility that trained immunity might represent another interesting mechanism to study and to exploit for AIT protocols. Clinical studies in children with allergic asthma have shown that HDM-AIT increased methylation in the *Il4* gene promoter and reduced IL-4 production by PBMCs compared with those in untreated children [66]. The identification of tolerance inducers in the context of allergic asthma is essential for advancing the design of more effective AIT strategies for disease management. In this context, allergoid-mannan conjugates represent a promising approach because they target dendritic cells and induce them to produce IL-10. These conjugates can also reprogram monocytes to differentiate into tolerogenic dendritic cells [67] and have demonstrated safety and efficacy in different phase 2 trials involving different allergens [68–70]. Allergoid-mannan conjugates have been shown to shift dendritic cells metabolism from glycolysis to increased oxidative phosphorylation while also enhancing histone activation marks at the promoters of anti-inflammatory genes such as IL-10 [67]. This finding strongly suggests that allergoid-mannan conjugates can induce epigenetic changes in innate immune cells, contributing to a long-term anti-inflammatory response, and therefore represent a future strategy for AIT.

### Biologics in asthma management

Although allergen-specific AIT has demonstrated efficacy in many patients with asthma, especially those with confirmed allergen sensitization, others unfortunately have shown suboptimal responses or cannot benefit from AIT because they have no signs of allergic sensitization. For these patients, especially those with difficult-to-treat asthma, biologics have become an important therapeutic alternative (Table 1).

**Table 1:** Biologic agents approved for the treatment of severe asthma

Target	Biologics	Mechanism of action	Patients	Clinical outcomes
**IgE**	Omalizumab	Reduces circulating IgE levels and therefore limits mast cell and basophil activation	Moderate to severe uncontrolled asthma	Improved lung function, decreased exacerbation rates
**IL-5**	Mepolizumab, Reslizumab	Prevents activation and survival of eosinophils	Severe eosinophilic asthma	Improved lung function, decreased exacerbation rates, and corticosteroid usage
**IL-5Ra**	Benralizumab	Depletes eosinophils	Severe eosinophilic asthma	Improved lung function, decreased exacerbation rates, and corticosteroid usage
**IL-4Ra**	Dupilumab	Inhibits both IL-4 and IL-13 signaling in immune and non-immune cells	Moderate to severe uncontrolled eosinophilic asthma	Improved lung function, decreased exacerbation rates, and corticosteroid usage
**Tezepelumab**	TSLP	Reduces type 2 immune responses	Severe asthma not responding to high doses of corticosteroids	Decreased exacerbation rate

#### Biologics targeting T2^high^ asthma

##### Targeting IgE in T2^high^ asthma

**Fig. 3:**
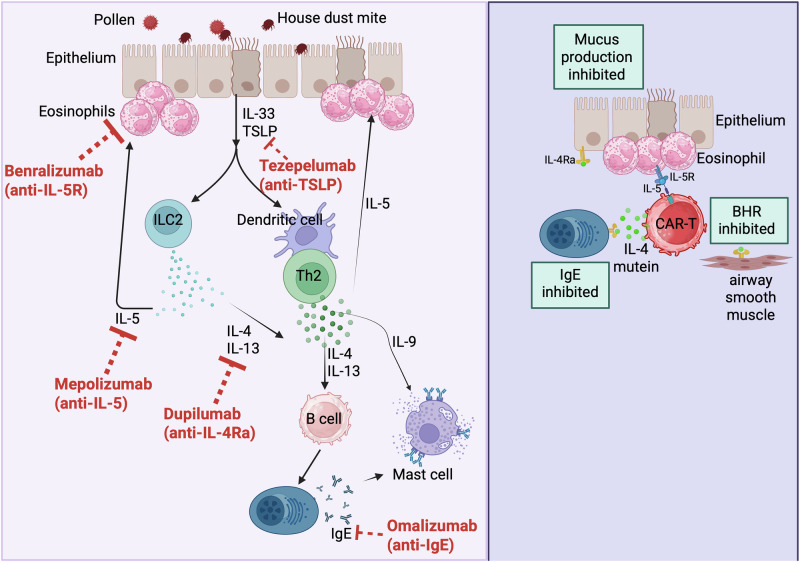
Targets of currently used biologics and future CAR-T-cell use to treat asthma. Left side: Exposure of patients with T2high asthma to allergens or pollutants triggers the release of the alarmins IL-33 and TSLP by epithelial cells. These cytokines activate ILC2s to release Th2 cytokines (IL-4, IL-5, and IL-13) and activate lung dendritic cells to induce and maintain Th2 cell-associated responses. Th2 cytokines (IL-4, IL-,5 and IL-13) drive IgE isotype switching in B cells, eosinophilic inflammation, mucus hypersecretion, and bronchoconstriction. Different biologics are currently used to block different mediators. Right side: Future therapies include CAR-T cells that can be engineered to be cytotoxic to IL-5R-expressing cells and to secrete an IL-4 mutation that can bind and block IL-4Rα signaling in several target cells. Such therapy inhibits airway eosinophilia, mucus production by epithelial cells, bronchial hyperresponsiveness (BHR), and IgE production

Omalizumab was first approved in 2003 and was the first biologic agent used to treat asthma. It is a monoclonal antibody that binds to IgE and prevents its binding to FcεRI on plasmacytoid dendritic cells, mast cells, basophils, and eosinophils. This then leads to the elimination of the IgE-omalizumab complexes from the circulation, decreasing both IgE levels and FcεRI surface expression (Fig. 3). The efficacy of omalizumab has been demonstrated in clinical trials as reducing the doses of corticosteroids needed to control the disease, improving lung function, and reducing exacerbation rates [71–74]. The link between IgE and virus-induced exacerbations might seem unexpected, but it can be explained by the fact that IgE is able to impair plasmacytoid DC functions when engaging FcεRI on their surface. Plasmacytoid DCs are crucial sources of type I IFNs and play a central role in antiviral responses. By binding to FcεRI on plasmacytoid DCs, IgE is able to reduce intracellular type 1 IFN signaling [75]. Such mechanisms could have detrimental effects upon encountering respiratory viruses, during which type I IFNs are crucial for viral clearance. In addition, FcεRI-IgE crosslinking on human plasmacytoid DCs can hamper their capacity to induce regulatory T cells [76]. When omalizumab or ligelizumab, an anti-IgE antibody with higher affinity than omalizumab, is administered, and as the levels of IgE decrease, both the antiviral and tolerogenic functions of plasmacytoid DCs can be restored, contributing to the generation of functional regulatory T cells, the restoration of type I IFN production, and the prevention of exacerbations [76–78]. Omalizumab is usually given to patients with severe allergic T2^high^ asthma with elevated serum IgE levels, multiple sensitizations, elevated FeNO, and blood eosinophilia > 300/μL [79]. Only approximately 70% of children and adults treated with omalizumab show good to excellent responses to treatment [80], and anti-IgE treatment is associated with a 44% decrease in severe exacerbations [81], highlighting the need for additional treatment options for those who do not respond.

##### Targeting IL-5 in T2^high^ asthma

IL-5 is a very potent mediator of the inflammatory cascade in allergic responses. The binding of IL-5 to its receptor, IL-5R, drives eosinophil maturation in the bone marrow as well as their activation and survival in inflamed tissues. IL-5 enhances the release of mediators from eosinophils and, as such, contributes to eosinophil-induced tissue damage, airway inflammation, and asthma exacerbation [82]. Because eosinophils are central to the pathophysiology of asthma, they are considered important targets for therapeutic interventions (Fig. 3). In murine studies, the absence of IL-5 in deficient animals or the blockade of IL-5 with monoclonal antibodies led to improved symptoms of ovalbumin- or HDM-induced asthma, including airway remodeling, airway hyperresponsiveness and mucus production by airway epithelial cells [83, 84]. In humans, three main biologics directed toward IL-5 (mepolizumab, reslizumab) IL-5R (benralizumab) have been designed and used as add-on treatments for severe asthma patients with eosinophilic inflammation (Table 1). These therapies have been shown to effectively reduce asthma exacerbations as well as the use of systemic corticosteroids in patients with severe asthma [85, 86]. Further analysis revealed that patients with higher blood eosinophil counts had a greater response in terms of decreased exacerbation numbers [87, 88]. In addition, benralizumab was shown to improve the mucus score and ventilation defects of severe asthma patients who presented with persisting mucus plugs in their airways [89]. The pooled rate of clinical remission under biologics is between 30% and 40%, depending on the criteria used to define remission [90]. The ultimate goal of any treatment is to achieve clinical remission. A recent real-world study revealed that most patients receiving benralizumab achieved partial or complete clinical remission, whereas this remission was clearly less common when mepolizumab was used [91, 92]. However, it is difficult to compare the effects of the 2 biologics head-to-head because of the different patient characteristics and, more importantly, because of the difference in the criteria used to define clinical remission. More data on airway hyperresponsiveness are still needed for anti-IL-5 therapy, but a recent phase 4 clinical trial revealed reduced airway hyperresponsiveness in patients with severe eosinophilic asthma treated with benralizumab [93].

A recent study reported that patients with a significant decrease in FeNO levels early after the start of anti-IL-5 therapy had higher remission rates than FeNO nonresponders [94]. These findings have important consequences since they argue that early FeNO response to anti-IL-5 agents might represent a biomarker to guide management decisions with biologics toward remission of severe asthma. Although they are very effective, one major drawback of anti-IL-5 agents is that they need to be administered every 4 weeks. Last year, a study reported that Depemokimab, a biologic agent that is effective at 6-month dosing intervals, was able to decrease exacerbation rates in patients with severe eosinophilic asthma [95]. Future research and real-world data will be needed to validate the long-term efficacy of Depemokimab and its role in asthma management.

##### Targeting IL-4 and IL-13 in T2^high^ asthma

IL-4 and IL-13 play crucial roles in T2^high^ asthma because of their capacity to drive Th2 cell differentiation and IgE production and because these cytokines can directly influence lung structural cells, especially fibroblasts, smooth muscle cells, and epithelial cells, and, as such, contribute to features of remodeling (collagen deposition, smooth muscle cell thickening, and epithelial shedding). These changes ultimately lead to persistent symptoms, decreased airway hyperresponsiveness, and airflow limitation. IL-4 and IL-13, along with their receptors, have therefore become targets for add-on biological therapy in T2^high^ asthma patients.

The identification of IL-4 as a target in asthma stems from murine studies showing that the absence of the blockade of IL-4 prevents the development of airway inflammation and other asthma features [84]. Later, the design of a humanized monoclonal antibody directed against IL-4, called Pascolizumab, showed efficacy in blocking IL-4 bioactivity in several in vitro cultured human cell lines. Although the antibody showed safety in monkeys and humans, pascolizumab had an insignificant effect during phase 2 trials and was discontinued [96]. Another compound, altrakincept, which is an inhaled humanized recombinant IL-4R, was used to block endogenous IL-4 in patients with asthma. Despite initial promising effects on lung function in patients with mild asthma [97], the product was discontinued because of its lack of efficacy in phase 3 clinical trials [98]. These failures suggest that targeting IL-4 is not sufficient to improve clinical symptoms in asthmatic patients.

In parallel, strategies aimed at IL-13 blockade have been used with the hope that interfering with this cytokine would relieve patients with T2^high^ asthma symptoms. Several antibodies have been developed to block either the binding of IL-13 to its receptor, IL-4Rα/IL-13Rα1, or the downstream signaling of this receptor. Anrukinzumab, a humanized anti-IL-13 antibody, was used in patients with mild asthma, and although initial studies reported some increase in lung function, the antibody was discontinued because of its limited effectiveness in patients with uncontrolled asthma [99]. Lebrikizumab, another anti-IL-13 antibody, was able to improve lung function and reduce the levels of FeNO, an indicator of airway inflammation, in patients with mild asthma. Additionally, it was effective in reducing exacerbation rates, but only in patients with severe asthma exhibiting high levels of periostin, a biomarker associated with early T2^high^ asthma [100, 101]. However, other trials have shown mixed effects in patients with mild asthma, questioning the selection of patients and asthma heterogeneity in those selected patients. A reanalysis of patients with elevated FeNO and prior exacerbations revealed the efficacy of lebrikizumab in reducing asthma exacerbations [102]. One possibility for the lack of efficacy of Lebrikizumab in mild asthma patients might be that IL-13 may not be the cytokine responsible for the symptoms in such a patient population, and a role for IL-13 may be significant only in more severe forms of the disease [103]. One important limitation of lebrikizumab, however, is its immunogenic potential, with approximately 10% of patients in clinical trials developing anti-drug antibodies, which may limit treatment efficacy or safety [100, 101]. A third antibody targeting IL-13, tralokinumab, has undergone phase 1, 2 and 3 clinical trials in patients with severe uncontrolled asthma. However, this antibody failed to reduce exacerbations even if a positive effect was observed in patients with high periostin levels, which suggested the use of this protein as a biomarker for future patient selection [104].

Collectively, these findings suggest that targeting IL-4 or IL-13 individually is insufficient to achieve asthma control, highlighting the need for alternative strategies. Interestingly, IL-4 and IL-13 share a common receptor unit, IL-4Rα, making it a strategic target for dual pathway inhibition. Dupilumab, a human monoclonal antibody directed against IL-4Rα, inhibits both the IL-4- and IL-13-induced signaling pathways (Fig. 3). A first phase 2 study performed in moderate-to-severe asthmatics with high eosinophil counts revealed that dupilumab allowed less use of corticosteroids while at the same time improving lung function and reducing exacerbation rates [105]. The levels of the Th2-associated biomarkers FeNO, IgE, and eotaxin were decreased without affecting peripheral eosinophilia. Interestingly, dupilumab has been shown to have positive effects on patients with asthma regardless of eosinophil count [106]. Phase 3 trials reported an improvement in lung function, a reduction in exacerbation rates, an improvement in symptoms and quality of life and reduced glucocorticoid dependence [107–110]. Interestingly, similar beneficial effects were also recently reported in children with severe asthma [111]. It will be interesting to know how long the effect of dupilumab on lung function can be maintained over time. Recent data have suggested that dupilumab has an effect for 3 years [112], but whether the control of lung function would last even longer remains to be determined. Additionally, because IL-4Rα is expressed on different lung structural cells, it will be important to verify whether dupilumab use can also impact lung remodeling, a feature of asthma. To date, only 2 studies have reported effects on mucus secretion and airway wall thickness [113, 114]. Dupilumab decreased mucus plugging in type 2 severe asthmatic patients [115].

##### Targeting the epithelial-derived alarmins IL-33 and TSLP in T2^high^ asthma

A new arena in the development of biologics focuses on alarmins (mainly IL-33 and TSLP) because of the capacity of these epithelial-derived cytokines to initiate and maintain type 2 immune responses (Fig. 3). The development of biologics targeting alarmins is aimed at fulfilling the unmet needs of patients for whom other biologics (targeting IgE, IL-4Ra or IL-5) would not work to control their symptoms.

IL-33 is expressed not only by barrier epithelial cells [116] but also by other lung structural cells, including endothelial cells, fibroblasts, and airway smooth muscle cells [117, 118]. IL-33 can be released passively upon tissue injury but can also be induced after proteolytic allergen exposure [119, 120]. Preclinical models have shown that IL-33 is upstream of the ILC2 and Th2 cascades, leading to eosinophil and neutrophilic inflammation [121, 122]. In addition, IL-33 can directly activate mast cells to release high levels of the type 2 cytokines IL-5 and IL-13 [123]. Different biologics directed against IL-33 or its receptor, ST2, have been developed and are currently being tested in clinical trials. Itepekimab, developed by Regeneron, failed to demonstrate superiority over dupilumab but was, however, able to reduce the loss of control of the disease compared with a placebo [124]. Astegolimab, an anti-ST2 antibody developed by Genentech, significantly reduced the asthma exacerbation rate in a phase 2b study [125]. Tozorakimab, an anti-IL-33 antibody developed by AstraZeneca, is currently being tested in phase 2a asthma patients, but the results are not yet available. However, one important aspect to consider is that the targeting of IL-33 or ST2 seems to improve patients with lower blood eosinophils [125]. If this is confirmed in all clinical trials, this should allow for a better choice of patients to be treated with such biologics.

TSLP is also considered an alarmin, and lung epithelial cells, as well as immune cells (dendritic cells, basophils, and mast cells) release TSLP in response to air pollutants, respiratory viruses, or allergens [126]. In response to TSLP, dendritic cells can drive the differentiation of Th2 cells in humans and mouse models [127, 128]. TSLP can enhance type 2 immunity through its capacity to activate eosinophils to release extracellular traps [129] but can also activate ILC2s, B cells, basophils, mast cells, monocytes, smooth muscle cells, or fibroblasts. In patients with asthma, elevated TSLP expression has been reported in the bronchial mucosa compared with that in healthy individuals, and high TSLP levels have been correlated with increased asthma severity [130–132]. As such, it is not surprising that the blockade of TSLP has been envisaged as a therapeutic strategy in asthma. To date, clinical data concerning asthma are available for only one anti-TSLP therapy, tezepelumab. Tezepelumab was approved as an add-on treatment for severe asthma, irrespective of blood eosinophil count. The phase 3 results of the clinical studies show clear efficacy for reducing asthma exacerbations in patients with high blood eosinophil counts and/or FeNO levels, both of which are markers of T2^high^ inflammation. In addition, compared with the placebo, tezepelumab was accompanied by a strong reduction in the number of tissue eosinophils and an improvement in lung function, presumably via effects on mast cells and smooth muscle cells [133, 134]. Finally, tezepelumab was able to significantly reduce mucus plug scores, mainly in patients with high blood eosinophil counts and elevated FeNO levels [135]. This may be one of the most important effects of TSLP blockade. Indeed, mucus plugs are tenacious, very persistent, and present in patients with the most severe forms of T2^high^ asthma and greater lung function deficits [136]. There is currently no treatment that can remove these plugs; therefore, mucus plugging of the airways is now considered one of the greatest unmet medical needs of clinical asthma care. It remains to be seen whether all the beneficial effects of tezepelumab, including those on mucus plugs, persist after biological cessation, making this TSLP-targeting antibody a true disease-modifying drug in asthma. Very recently, the results of a phase 1b clinical trial using another TSLP-targeting therapy reported that patients with asthma treated with Verekitug (a potent long-acting anti-TSLP receptor antibody that only requires a 6-month dosing) had decreased Feno levels and blood eosinophil counts 24 weeks after biologic cessation (reviewed in [137]). Phase 2 clinical trials are ongoing and it remains to be seen whether this long-acting biological agent also has effects on other asthma symptoms.

### Biologics targeting T2^low^ asthma

While the molecular pathways involved in T2^high^ asthma have been extensively studied and are now better understood, the ones responsible for T2^low^ asthma are less well characterised. T2^low^ asthma generally encompasses patients with neutrophilic asthma and is usually more resistant to corticosteroid therapy. Because the root mechanisms of T2^low^ asthma are poorly understood, only a few biologics have been developed and used in T2^low^ asthma patients.

#### Targeting neutrophils in T2^low^ asthma

Because T2^low^ asthma patients are characterized by neutrophilic inflammation, IL-17 has attracted interest as a potential target in these patients. Secukinumab, a human antibody that targets IL-17A, and brodalumab, an antibody that targets IL-17RA, have been developed and tested in severe uncontrolled asthma. Both compounds have been discontinued because of their lack of efficacy [138]. The failure of these clinical studies might be explained by the fact that, at the time, the neutrophil counts were not used as an inclusion criterion, and it is therefore likely that the patients selected were heterogeneous with respect to neutrophil numbers. Because the recruitment of neutrophils to sites of inflammation involves CXCR2, a high-affinity receptor for IL-8, CXCR2 antagonists have been developed and tested as an add-on to standard therapy in patients with persistent uncontrolled asthma. Despite being able to reduce neutrophil counts in the blood, the CXCR2 antagonist used failed to reduce exacerbation rates or any other clinical outcomes measured [139]. One important limitation of this study was that the authors did not use a neutrophilic marker of airway inflammation and, as such, were not sure whether the patients used truly had neutrophilic asthma. This study failed to investigate the contribution of neutrophils to the pathobiology of asthma severity or exacerbation in the absence of infectious agents.

#### Targeting the alarmin TSLP in T2^low^ asthma

TSLP is certainly one of the most promising targets for treating T2^low^ asthma. This finding was quite surprising because this target was initially thought to benefit mainly patients with T2^high^ asthma since TSLP has been classically associated with type 2 immunity [127, 128]. However, the blockade of TSLP in T2^low^ patients improved their lung function [133, 140], but this effect was less strong than that observed in T2^high^ patients [140]. In addition to affecting lung function, tezepelumab has been shown to significantly reduce exacerbation rates by 62-71% in severe patients with uncontrolled asthma, who are classified with a T2^low^ disease form (low IgE levels and low eosinophil counts) [141]. How TSLP blockade can lead to improvements in lung function in T2^low^ patients is still unclear. Since TSLP can directly activate mast cells and smooth muscle cells, the reduction in airway hyperresponsiveness in T2^low^ patients may be due to reduced activation of these cell types in a T2-independent manner. In addition, 82% of patients with low blood eosinophil counts classified as T2^low^ still presented some degree of T2 airway inflammation, with the presence of airway submucosal eosinophilia [10, 142]. This residual T2 component, possibly with the help of TSLP, may contribute to the pathophysiology of the disease in T2^low^ patients. Future studies are needed to understand the role of TSLP in T2^low^ asthma.

#### Chimeric antigen receptor (CAR)-cells: the future of asthma immunotherapy?

To date, AIT and biologics have proven effective in treating asthma. However, both types of immunotherapies have advantages and disadvantages. Indeed, AIT has been shown to be a disease modifier, as it can restore homeostasis in patients with allergic asthma, but the treatment is very long and cannot be offered to severe asthma patients because of adverse effects. Biologics can benefit many patients with asthma, but the disease usually returns when the treatment is stopped. Therefore, new forms of therapies that can cure patients after cessation are needed.

Recently, the use of CAR-T cells has attracted much interest in the field of asthma. CARs are recombinant receptors that target a specific antigen. The best-known CAR-T cells are those used for hematological malignancies, solid tumors, and some inflammatory diseases, such as autoimmune diseases [143]. Preclinical models of allergic asthma have shown that CAR-Tregs are more efficient than conventional Tregs in reducing several features of allergic asthma, including eosinophilic airway inflammation and airway hyperresponsiveness [144]. A very recent study reported the possibility of using CAR-T cells targeting IL-5R^+^ eosinophils as a cure for asthma [145]. In this elegant study, the capacity of CAR-T cells to kill eosinophils was similar to that of benralizumab, but at the same time, these CAR-T cells were designed to continuously secrete an IL-4 mutein that binds to IL-4Rα, the receptor for IL-4 and IL-13, and, as such, behaves like dupilumab, a blocking anti-IL-4Ra antibody used in asthma. The use of these CAR-T cells by Jin et al [145] led to a decrease in eosinophilia and a long-lasting decrease in inflammation and asthma symptoms. CAR-T cells could therefore represent a revolutionary treatment for asthma. One major drawback of CAR-T-cell therapy in inflammatory diseases is preconditioning with ablative chemotherapeutics, which are required to infuse these cells, allowing their survival in patients. Such treatment is heavy, especially for patients with non-life-threatening diseases such as asthma. One way to circumvent the use of chemotherapy would be to find strategies to allow the engraftment of CAR-T cells in patients without preconditioning. Jin et al [145] reported that the CRISPR-guided deletion of specific genes endowed them with stemness and allowed the transfer of these cells without preconditioning. In turn, a single infusion of CAR-T cells protected the mice from asthma for up to 1 year after transfer. After killing eosinophils, CAR-T cells enter a metabolically dormant state but persist in vivo [145]. It will be interesting to identify the survival factors required for their long-term residency in their niche. Although the strategy employed by Jin et al. is very appealing, it remains to be seen whether it can be used or tailored for use in the clinic, especially for inflammatory diseases such as asthma.

## Conclusions

Although the clinical efficacy of AIT is widely recognized, it remains inefficient in many patients. In addition, AIT can cause serious adverse effects, which is a major obstacle to its wider clinical application. The recent introduction of biologics for the management of asthma has changed the lives of many patients. Initially, used as monotherapies, these biologics are now being explored as combination therapies with AIT. Future studies will determine whether such combined therapy is safe and presents long-term immunological and clinical benefits. In addition, the recognition that some patients with T2^low^ asthma have a T2 component certainly opens new perspectives in potential treatments for these difficult-to-treat patients. Furthermore, many of the T2 biologics used in asthma may also be used for other indications. Indeed, recently, a T2 component (elevated sputum eosinophil counts) has also been identified in a subset of patients with chronic obstructive pulmonary disease (COPD) [146]. Dupilumab was able to improve lung function and reduce exacerbation rates in COPD patients with high blood eosinophil counts, but to a lesser extent than what is achieved in asthmatic patients [147]. Promising results have also been obtained with IL-33 blockade [148], and given the success in asthma, the results with tezepelumab in COPD patients are awaited.

Finally, all the clinical trials performed thus far have included only adults and older children. No biologics are currently approved for children under 6 years of age, mainly because of diagnostic challenges. Indeed, differentiating severe asthma from viral-induced wheezing is difficult in preschool children. It is also likely that the immune mechanisms involved in early childhood asthma may differ from those of older children, requiring tailored therapeutic targets [149]. Clinical trials in younger children are therefore needed to assess the efficacy and use of biologics in this age group.
